# Predicting the risk of subclinical atherosclerosis based on interpretable machine models in a Chinese T2DM population

**DOI:** 10.3389/fendo.2024.1332982

**Published:** 2024-02-27

**Authors:** Ximisinuer Tusongtuoheti, Yimeng Shu, Guoqing Huang, Yushan Mao

**Affiliations:** ^1^ Department of Endocrinology, The First Affiliated Hospital of Ningbo University, Ningbo University, Ningbo, China; ^2^ Health Science Center, Ningbo University, Ningbo, China

**Keywords:** subclinical atherosclerosis, type 2 diabetes mellitus, independent risk factors, interpretable machine learning, prediction model

## Abstract

**Background:**

Cardiovascular disease (CVD) has emerged as a global public health concern. Identifying and preventing subclinical atherosclerosis (SCAS), an early indicator of CVD, is critical for improving cardiovascular outcomes. This study aimed to construct interpretable machine learning models for predicting SCAS risk in type 2 diabetes mellitus (T2DM) patients.

**Methods:**

This study included 3084 T2DM individuals who received health care at Zhenhai Lianhua Hospital, Ningbo, China, from January 2018 to December 2022. The least absolute shrinkage and selection operator combined with random forest-recursive feature elimination were used to screen for characteristic variables. Linear discriminant analysis, logistic regression, Naive Bayes, random forest, support vector machine, and extreme gradient boosting were employed in constructing risk prediction models for SCAS in T2DM patients. The area under the receiver operating characteristic curve (AUC) was employed to assess the predictive capacity of the model through 10-fold cross-validation. Additionally, the SHapley Additive exPlanations were utilized to interpret the best-performing model.

**Results:**

The percentage of SCAS was 38.46% (n=1186) in the study population. Fourteen variables, including age, white blood cell count, and basophil count, were identified as independent risk factors for SCAS. Nine predictors, including age, albumin, and total protein, were screened for the construction of risk prediction models. After validation, the random forest model exhibited the best clinical predictive value in the training set with an AUC of 0.729 (95% CI: 0.709-0.749), and it also demonstrated good predictive value in the internal validation set [AUC: 0.715 (95% CI: 0.688-0.742)]. The model interpretation revealed that age, albumin, total protein, total cholesterol, and serum creatinine were the top five variables contributing to the prediction model.

**Conclusion:**

The construction of SCAS risk models based on the Chinese T2DM population contributes to its early prevention and intervention, which would reduce the incidence of adverse cardiovascular prognostic events.

## 1 Introduction

Type 2 diabetes mellitus (T2DM) is a metabolic disorder characterized by insulin resistance and relative insulin deficiency. In recent years, the prevalence of T2DM has increased steadily, which has become a serious public health issue. Updated estimates for 2021 showed that about 10.5% of the global population had T2DM, a prediction that this figure would increase to 12.2% by 2045 (1). Cardiovascular disease (CVD) is the leading cause of death and disability in T2DM (2, 3). Studies have shown that the risk of CVD in patients with T2DM is two to four times higher than in individuals without diabetes (4, 5). Atherosclerosis (AS), the predominant pathophysiologic process in CVD, may begin early in life and remain latent and asymptomatic for extended periods before progressing to advanced stages. Subclinical atherosclerosis (SCAS) serves as an early indicator of atherosclerotic burden, and its timely recognition can help slow down or prevent the progression to CVD (6). Therefore, the early identification and effective management of SCAS in individuals with T2DM are crucial strategies to mitigate progression to overt CVD, thereby improving life expectancy and quality.

Diagnostic methods for SCAS include angiography, intravascular ultrasound, carotid ultrasound (CUS), computed tomography (CT), and magnetic resonance imaging. Measuring carotid intima-media thickness (CIMT) and coronary artery calcification (CAC) using CUS and CT has become the mainstay for assessing SCAS, owing to their noninvasive and easily accessible nature (7, 8). However, large-scale use of CUS and CT could inevitably lead to the waste of medical resources and increased costs. Thus, establishing an assessment tool capable of screening individuals at high risk for SCAS without the need for imaging examinations is of great significance.

In recent years, artificial intelligence (AI) and machine learning (ML) have increasingly been utilized in the healthcare field (9). Several studies currently employ ML methods to research SCAS. For example, Sánchez-Cabo et al. (10) developed a SCAS risk prediction model for young asymptomatic individuals using four ML algorithms, demonstrating good clinical predictive value with an area under the receiver operating characteristic curve (AUC) of 0.890. Additionally, Núñez et al. (11) used ML methods to identify circulating proteins that can predict SCAS, also showing good clinical predictive value with an AUC of 0.730. However, there are few reports on the risk prediction models for SCAS in T2DM patients. The purpose of this study was to establish SCAS risk prediction models based on interpretable machine learning algorithms, contributing to the early identification of SCAS and guiding appropriate prevention and interventions.

## 2 Methods

### 2.1 Participants

**Figure 1:**
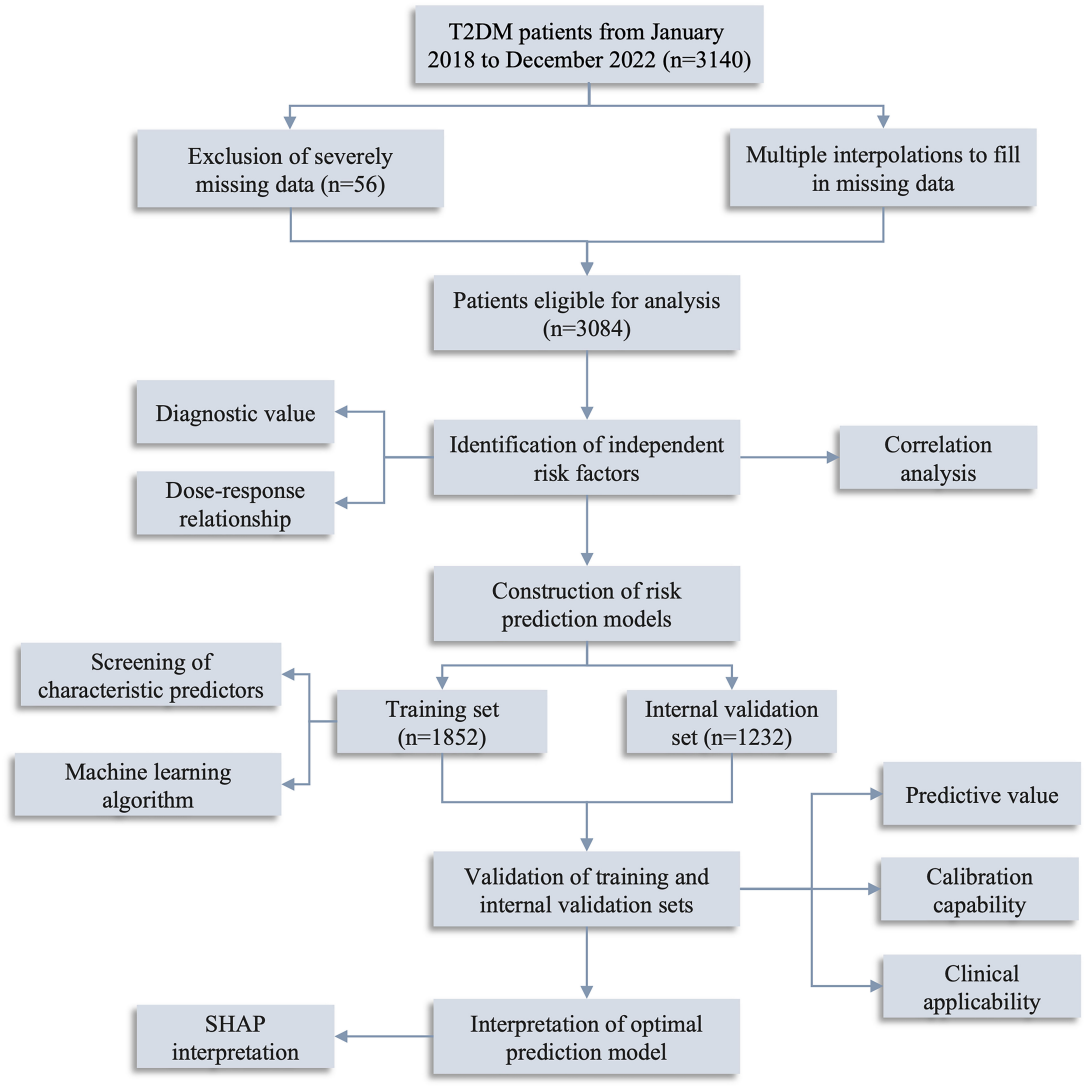
Flow diagram of the study. T2DM, type 2 diabetes mellitus; SHAP, Shapley Additive exPlanations.

This study enrolled 3140 T2DM individuals who had sought medical care through outpatient visits, inpatient admissions, and routine physical examinations at Zhenhai Lianhua Hospital in Ningbo, China, from January 2018 to December 2022. The sample size for this study adhered to the rule of 10 events per variable (12). The demographic data, comorbidities, complications, and biochemical parameters were obtained by questionnaires and laboratory tests. Inclusion criteria: participants aged ≥ 18 years who either self-report T2DM, are undergoing pharmacological treatment for T2DM, or meet the diagnostic criteria of T2DM. These criteria include fasting blood glucose (FBG) levels of ≥ 7.0 mmol/L, 2-hour blood glucose levels of ≥ 11.1 mmol/L, or a glycated hemoglobin level of ≥ 6.5% (13). Exclusion criteria: individuals with other forms of diabetes mellitus, concurrent coronary heart disease or cerebral infarction, acute complications related to diabetes mellitus, malignant tumors, severe liver and kidney function abnormalities, or pregnancy. SCAS was defined as CIMT > 1.0 mm and/or the presence of plaque without clinical manifestations (14). Data with more than 20% missing were excluded (n=56), and those with less than 20% were filled by multiple interpolations (**Supplementary Figure 1**). Ultimately, 3084 T2DM patients were included in this study. The study’s flow diagram is depicted in **Figure 1**.

### 2.2 Clinical baseline data

Participants’ general characteristics include gender, age, body mass index, and blood pressure (both systolic and diastolic measurements). Blood cell counts comprise white blood cell count (WBC), neutrophil count, eosinophil count, basophil count (BASO), lymphocyte count (LYC), red blood cell count, hemoglobin, red blood cell distribution width, mean red blood cell volume (MCV), platelet count, platelet distribution width (PDW), and mean platelet volume (MPV). Biochemical indicators encompass total cholesterol (TC), triglycerides, high-density lipoprotein (HDL), low-density lipoprotein (LDL), FBG, total protein (TP), albumin (ALB), aspartate aminotransferase, alanine aminotransferase, gamma-glutamyl transpeptidase (GGT), serum uric acid (SUA), and serum creatinine (SCR).

### 2.3 Statistical analysis

Kolmogorov-Smirnov assessed sample distribution normality. Normal continuous variables were expressed as means (standard deviation, SD), non-normal continuous variables as median (interquartile range, IQR), and categorical variables as frequency (percentage, %). Between-group analyses involved independent samples t-tests for normal continuous variables, Mann-Whitney U tests for non-normal continuous variables, and chi-square tests for categorical variables. Box plots were used to elucidate the relationship between various metabolic parameters [including atherogenic index of plasma (AIP), Castelli risk index (CRI), metabolic score for insulin resistance (METS-IR), and triglyceride-glucose (TyG) index] and SCAS. The formulas for these parameters were calculated as follows: AIP = Log(TG/HDL); CRI = TC/HDL; METS-IR = Ln((2 * FBG + TG) * BMI)/(Ln(HDL)); TyG = Ln[(TG * FBG)/2]. Multivariate logistic regression identified independent risk factors for SCAS. Restricted cubic spline was employed to analyze the dose-response relationship betweent AIP and SCAS.

Least absolute shrinkage and selection operator (LASSO) combined with random forest-recursive feature elimination (RF-RFE) were used to screen for characteristic variables. Six ML methods, including linear discriminant analysis (LDA), logistic regression (LR), Naive Bayes (NB), random forest (RF), support vector machine (SVM), and extreme gradient boosting (XGboost), were used to model construction. The primary parameters used to evaluate the effectiveness of risk prediction models included accuracy, sensitivity, specificity, precision, recall, and the F1 score. AUC was utilized to assess the models’ predictive ability. Calibration curves and the Brier score were used to assess calibration capability, while decision curve analysis (DCA) was employed to evaluate clinical applicability. Additionally, the Shapley Additive exPlanations (SHAP) was used to interpret the best predictive model.

All statistical analyses were conducted using Python (https://www.python.org/, version: 3.9.0) and R (https://cran.r-project.org/, version: 4.1.3). All tests were two-sided and *P* < 0.05 was deemed statistically significant.

## 3 Results

### 3.1 Clinical baseline information of the study population

A total of 3084 participants were enrolled in this study, comprising 1898 individuals with T2DM without SCAS, and 1186 individuals with T2DM with SCAS. The percentage of SCAS in the T2DM population was found to be as high as 38.46%. The median age of participants was 56 years (IQR: 49-61). Participants in the SCAS group were older, with a median age of 58 years (IQR: 53-62), compared to 54 years (IQR: 46-60) in the control group. The male proportion was similar in both groups (74.6% in the SCAS group vs. 73.8% in the control group, *P* > 0.05). Additionally, statistically significant differences were observed between the groups in terms of routine blood tests, lipid and glucose levels, and liver and kidney function (*P* < 0.05). The baseline clinical characteristics of the study population are presented in **Table 1**.

**Table 1:** Univariate analysis of subclinical atherosclerosis.

	Overall	Normal	SCAS	*P*-value
N	3084	1898	1186	
Sex (male), %	2291 (74.3)	1416 (74.6)	875 (73.8)	0.639
Age, years	56.00 (49.00, 61.00)	54.00 (46.00, 60.00)	58.00 (53.00, 62.00)	<0.001
BMI, kg/m2	24.62 (22.72, 26.99)	24.69 (22.77, 26.99)	24.54 (22.68, 26.96)	0.209
SBP, mmHg	133.69 (17.48)	133.43 (16.70)	134.10 (18.65)	0.298
DBP, mmHg	81.00 (73.00, 89.00)	81.00 (74.00, 89.00)	80.00 (72.00, 89.00)	0.009
WBC, 10^9^/L	6.54 (1.73)	6.48 (1.67)	6.63 (1.81)	0.026
NEU, 10^9^/L	3.70 (2.97, 4.60)	3.60 (2.90, 4.50)	3.80 (3.00, 4.81)	<0.001
EOS, 10^9^/L	0.11 (0.07, 0.19)	0.11 (0.07, 0.19)	0.12 (0.07, 0.20)	0.123
BASO, 10^9^/L	0.02 (0.01, 0.03)	0.02 (0.01, 0.03)	0.02 (0.02, 0.03)	<0.001
LYC, 10^9^/L	2.00 (1.60, 2.50)	2.09 (1.60, 2.50)	1.92 (1.55, 2.40)	<0.001
RBC, 10^12^/L	4.88 (0.52)	4.93 (0.51)	4.79 (0.53)	<0.001
HB, g/L	150.00 (139.00, 159.00)	151.00 (140.00, 160.00)	148.00 (137.00, 158.00)	<0.001
RDW, %	12.50 (12.20, 12.90)	12.50 (12.20, 12.90)	12.60 (12.20, 13.00)	0.003
MCV, fL	91.00 (88.60, 94.00)	91.00 (88.10, 93.80)	91.90 (89.00, 94.90)	<0.001
PLT, 10^9^/L	226.93 (58.27)	227.49 (58.41)	226.04 (58.06)	0.499
PDW, %	15.00 (12.50, 16.30)	14.10 (12.30, 16.20)	15.90 (12.90, 16.30)	<0.001
MPV, fL	10.59 (1.15)	10.70 (1.15)	10.41 (1.13)	<0.001
TC, mmol/L	5.00 (1.16)	4.97 (1.07)	5.05 (1.30)	0.057
TG, mmol/L	1.50 (1.06, 2.21)	1.48 (1.04, 2.22)	1.54 (1.11, 2.20)	0.090
HDL, mmol/L	1.14 (0.95, 1.39)	1.16 (0.95, 1.44)	1.11 (0.96, 1.33)	0.001
LDL, mmol/L	2.82 (0.88)	2.80 (0.82)	2.85 (0.96)	0.081
FBG, mmol/L	6.80 (6.19, 8.35)	6.71 (6.18, 8.21)	6.98 (6.21, 8.61)	0.013
TP, g/L	73.00 (68.70, 76.60)	73.70 (70.10, 76.90)	71.50 (66.60, 75.50)	<0.001
ALB, g/L	44.90 (42.27, 46.60)	45.35 (43.10, 46.90)	44.00 (40.90, 46.00)	<0.001
AST, IU/L	23.00 (18.00, 29.00)	23.00 (18.00, 29.00)	23.00 (18.00, 30.00)	0.780
ALT, IU/L	24.00 (17.00, 38.00)	25.00 (17.00, 39.00)	23.00 (16.00, 37.00)	0.058
GGT, U/L	31.00 (21.00, 52.00)	30.00 (20.00, 51.00)	33.00 (22.00, 54.00)	0.008
SUA, μmol/L	357.19 (96.25)	354.05 (95.07)	362.22 (97.95)	0.022
SCR, μmol/L	66.00 (56.00, 76.00)	64.45 (55.10, 74.38)	68.00 (58.00, 79.00)	<0.001

BMI, body mass index; SBP, systolic blood pressure; DBP, diastolic blood pressure; WBC, white blood cell count; NEU, neutrophil count; EOC, eosinophil count; BASO, basophil count; LYC, lymphocyte count; RBC, red blood cell count; HB, hemoglobin; RDW, red blood cell distribution width; MCV, mean red blood cell volume; PLT, platelet count; PDW, platelet distribution width; MPV, mean platelet volume; TC, total cholesterol; TG, triglycerides; HDL, high-density lipoprotein; LDL, low-density lipoprotein; FBG, fasting blood glucose; TP, total protein; ALB, albumin; AST, aspartate aminotransferase; ALT, alanine aminotransferase; GGT, gamma-glutamyl transpeptidase; SUA, serum uric acid; SCR, serum creatinine.

**Figure 2:**
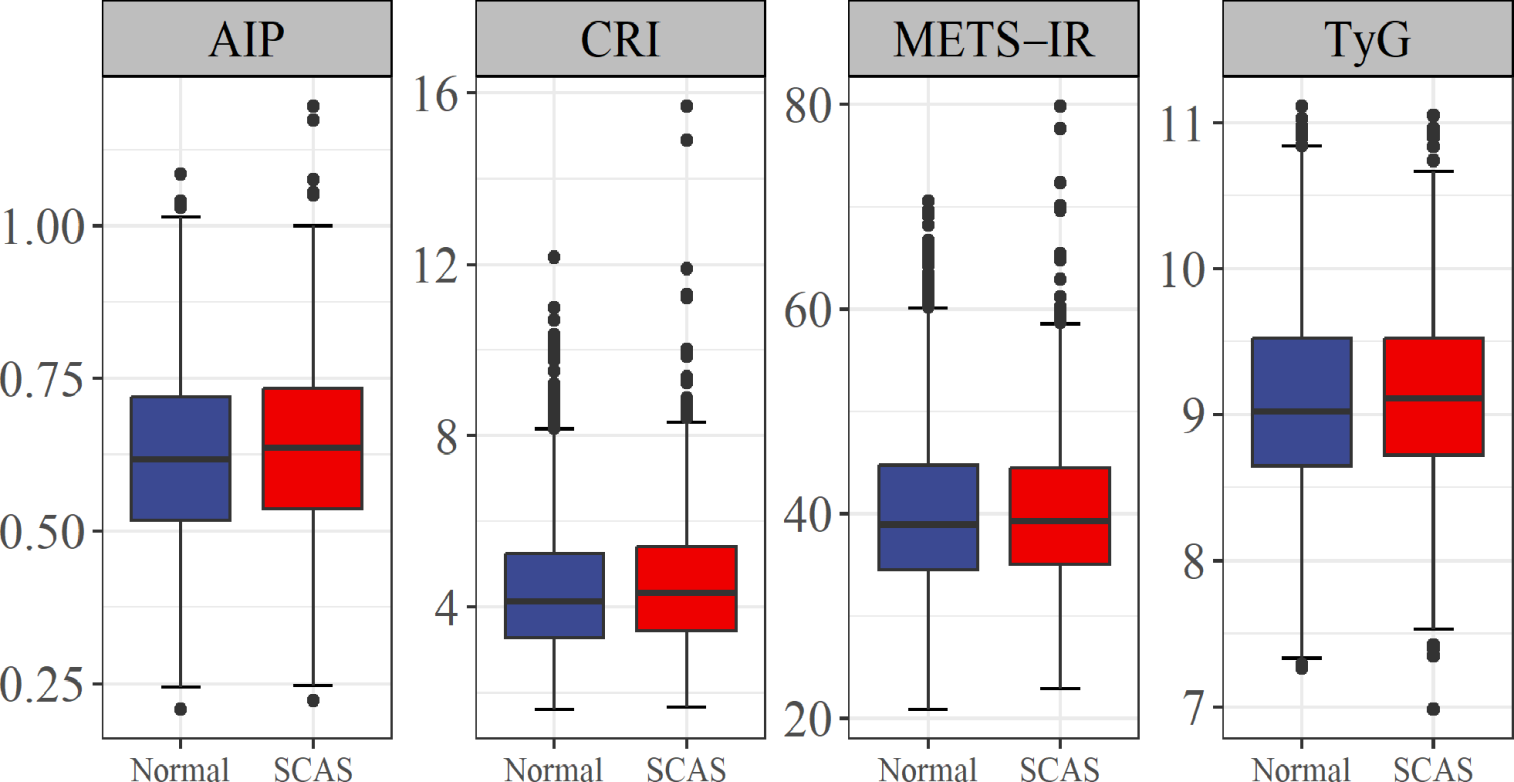
Association of four metabolism-related parameters with risk of SCAS. AIP, atherogenic index of plasma; CRI, Castelli risk index; TyG, triglyceride-glucose; METS-IR, metabolic score for insulin resistance; SCAS, subclinical atherosclerosis.

The AIP, CRI, METS-IR, and TyG index are metabolism-related parameters commonly used in the diagnosis and risk assessment of metabolism-related diseases (15–18). The current study showed that three metabolism-related parameters, including AIP, CRI, and TyG, were significantly higher in the SCAS group than in the control group (*P* < 0.05) (**Figure 2**).

### 3.2 Independent risk factors

**Figure 3:**
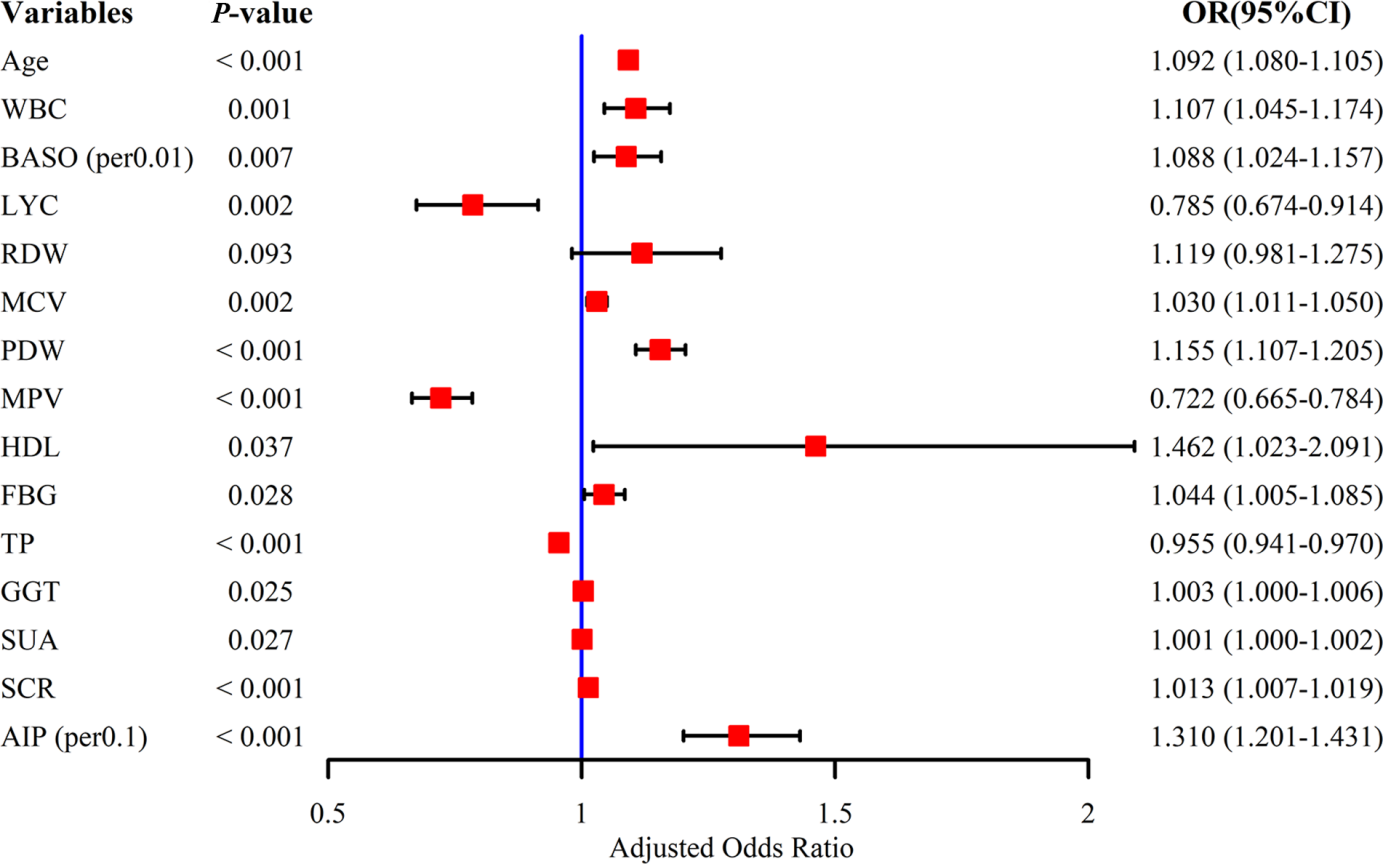
Multivariate logistic regression analysis of subclinical atherosclerosis. WBC, white blood cell count; BASO, basophil count; LYC, lymphocyte count; RDW, red blood cell distribution width; MCV, mean red blood cell volume; PDW, platelet distribution width; MPV, mean platelet volume; HDL, high-density lipoprotein; FBG, fasting blood glucose; TP, total protein; GGT, gamma-glutamyl transpeptidase; SUA, serum uric acid; SCR, serum creatinine; AIP, atherogenic index of plasma.

Nineteen potential risk factors associated with SCAS were initially screened by univariate analysis (*P* < 0.05) (**Table 1**). To ensure the accuracy and credibility of the findings, we calculated the variance inflation factor (VIF) for each variable and considered to exhibit lower multicollinearity when their VIF was below 10 (**Supplementary Figure 2**). Afterward, we performed stepwise backward logistic regression analysis with the Akaike information criterion to filter and remove multicollinear variables. Ultimately, fifteen variables were included in the multivariate logistic regression analysis, and the final fourteen variables such as Age, WBC, BASO, and LYC (*P* < 0.05) were identified as independent risk factors for SCAS (**Figure 3**).

**Figure 4:**
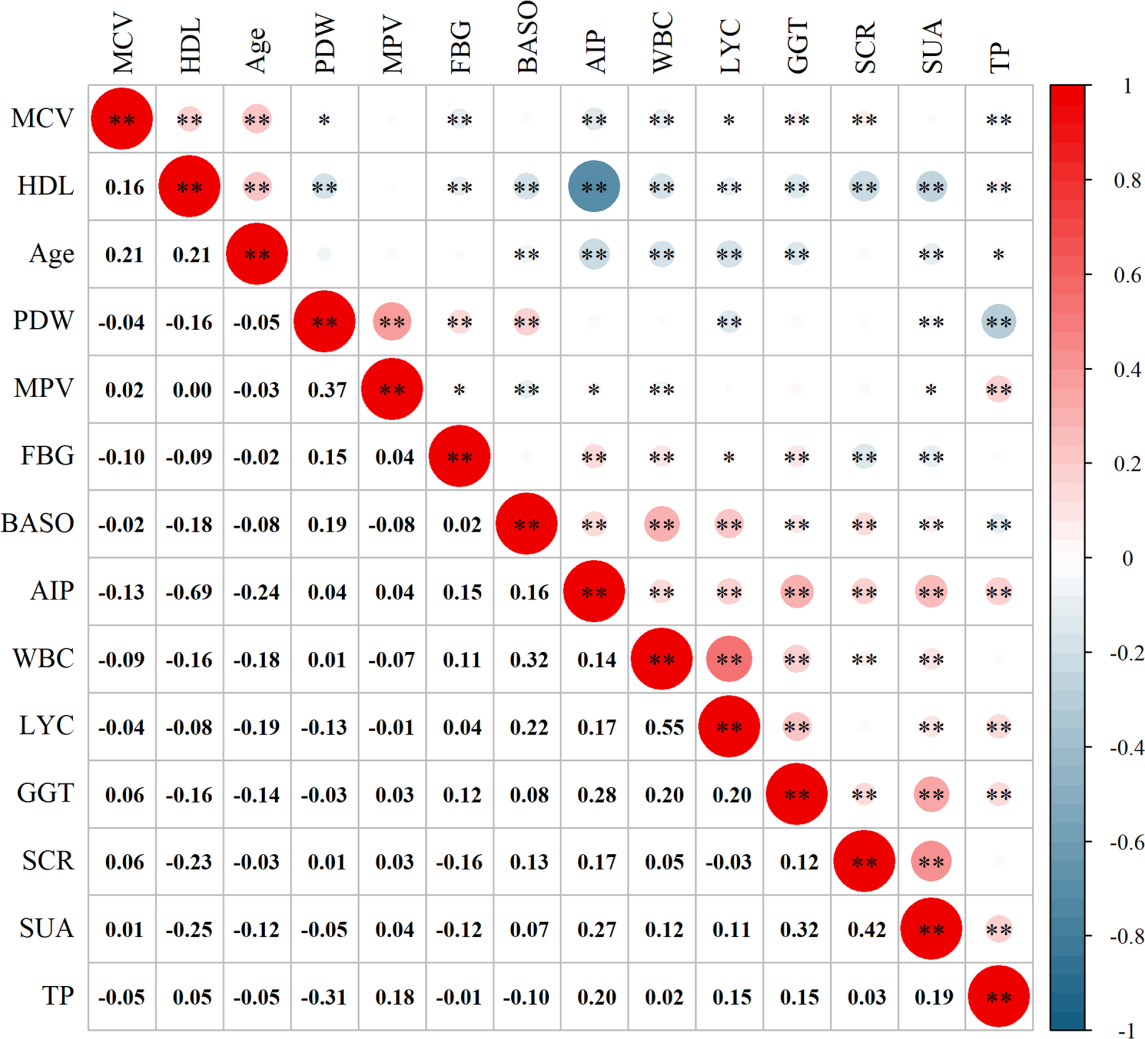
Correlation analysis between the variables. MCV, mean red blood cell volume; HDL, high-density lipoprotein; PDW, platelet distribution width; MPV, mean platelet volume; FBG, fasting blood glucose; BASO, basophil count; AIP, atherogenic index of plasma; WBC, white blood cell count; LYC, lymphocyte count; GGT, gamma-glutamyl transpeptidase; SCR, serum creatinine; SUA, serum uric acid; TP, total protein. *P < 0.05; **P < 0.01.

Based on the independent risk factors, we proceeded to explore the correlation between the variables (**Figure 4**). From the correlation analysis, we observed a negative correlation between AIP and Age (r = -0.24, *P* < 0.01), MCV (r = -0.13, *P* < 0.01), and HDL (r = -0.69, *P* < 0.01). Additionally, positive correlations were observed between AIP and WBC (r = 0.14, *P* < 0.01), GGT (r = 0.28, *P* < 0.01), and SUA (r = 0.27, *P* < 0.01).

**Figure 5:**
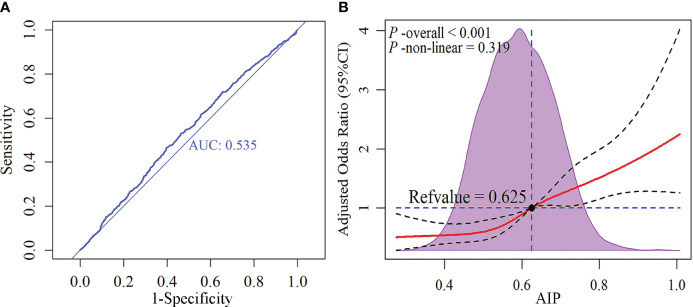
Receiver operating characteristic (ROC) curve and dose-response relationship between AIP and subclinical atherosclerosis. **(A)** ROC curve; **(B)** Dose-response relationship. AIP, atherogenic index of plasma.

To further assess the clinical applicability of AIP, we conducted a diagnostic experiment and a dose-response relationship study. The result of the diagnostic experiment (**Figure 5A**) revealed that although AIP holds promise as a potential biomarker for SCAS, its diagnostic value was moderate (AUC: 0.535). The dose-response relationship (**Figure 5B**) demonstrated a linear correlation between AIP and the risk of SCAS prevalence (*P*-overall < 0.001, *P*-non-linear = 0.319), with a significant increase in risk observed when AIP was greater than 0.625.

### 3.3 Construction of risk prediction models

**Figure 6:**
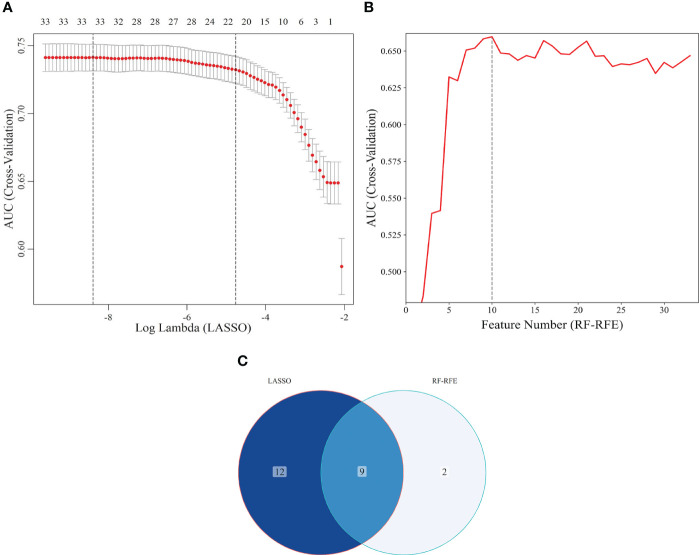
Screening of characteristic predictors. **(A)** Characteristic variables screening based on LASSO (lambda: 1SE); **(B)** Characteristic variables screening based on RF-RFE; **(C)** LASSO combined RF-RFE. LASSO, least absolute shrinkage and selection operator; SE, standard error; RF-RFE, random forest-recursive feature elimination.

The study population was divided into training and internal validation sets at a 6:4 ratio. The basic characteristics of the participants in the two sets did not differ (**Table 2**). LASSO enables a data dimensionality reduction algorithm that screens feature predictors by constructing a penalty function that compresses regression coefficients to zero (19). RF-RFE is a recursive backward feature elimination method that evaluates the importance of variables and progressively removes the least important ones, ultimately screening the optimal number of features (20). In the training set, LASSO combined with RF-RFE was applied to screen the most characteristic variables for SCAS (**Figures 6A, B**). Subsequently, the common variables screened by both algorithms were selected as predictors for constructing the SCAS risk prediction models, which included Age, FBG, TC, HDL, LDL, TP, ALB, SUA, and SCR (**Figure 6C**). To determine the optimal risk prediction model, six machine learning algorithms, namely LDA, LR, NB, RF, SVM, and XGboost, were employed to construct risk prediction models.

**Table 2:** Characteristics of participants in different sets.

	Training set	Internal validation set	*P*-value
N	1852	1232	
Sex (male), %	1367 (73.8)	924 (75.0)	0.486
Age, years	56.00 (49.00, 61.00)	55.00 (50.00, 61.00)	0.406
BMI, kg/m^2^	24.70 (22.81, 26.99)	24.56 (22.65, 26.95)	0.132
SBP, mmHg	133.83 (17.23)	133.48 (17.84)	0.590
DBP, mmHg	81.00 (73.00, 90.00)	81.00 (73.00, 89.00)	0.750
WBC, 10^9^/L	6.52 (1.73)	6.57 (1.72)	0.473
NEU, 10^9^/L	3.70 (2.96, 4.60)	3.70 (2.99, 4.67)	0.765
EOS, 10^9^/L	0.11 (0.07, 0.19)	0.11 (0.07, 0.19)	0.417
BASO, 10^9^/L	0.02 (0.01, 0.03)	0.02 (0.01, 0.03)	0.579
LYC, 10^9^/L	2.00 (1.60, 2.44)	2.00 (1.60, 2.50)	0.354
RBC, 10^12^/L	4.86 (0.53)	4.90 (0.52)	0.043
HB, g/L	150.00 (138.00, 159.00)	150.00 (139.00, 159.25)	0.124
RDW, %	12.60 (12.20, 13.00)	12.50 (12.20, 12.90)	0.113
MCV, fL	91.00 (88.80, 94.00)	91.00 (88.40, 94.00)	0.909
PLT, 10^9^/L	226.23 (59.25)	227.99 (56.77)	0.413
PDW, %	15.20 (12.50, 16.30)	14.60 (12.50, 16.30)	0.409
MPV, fL	10.59 (1.17)	10.58 (1.12)	0.726
TC, mmol/L	4.99 (1.16)	5.02 (1.17)	0.552
TG, mmol/L	1.50 (1.08, 2.20)	1.49 (1.03, 2.22)	0.288
HDL, mmol/L	1.14 (0.95, 1.39)	1.14 (0.97, 1.40)	0.254
LDL, mmol/L	2.81 (0.88)	2.83 (0.88)	0.622
FBG, mmol/L	6.78 (6.18, 8.36)	6.84 (6.20, 8.31)	0.433
TP, g/L	72.90 (68.60, 76.60)	73.00 (68.80, 76.50)	0.870
ALB, g/L	44.90 (42.30, 46.60)	44.85 (42.20, 46.52)	0.818
AST, IU/L	23.00 (18.00, 29.00)	23.00 (18.00, 30.00)	0.216
ALT, IU/L	24.00 (17.00, 37.00)	24.00 (17.00, 40.00)	0.098
GGT, U/L	31.00 (21.00, 50.00)	31.00 (21.00, 54.00)	0.319
SUA, μmol/L	356.67 (94.60)	357.97 (98.72)	0.714
SCR, μmol/L	65.90 (56.00, 76.00)	66.00 (56.00, 76.43)	0.864
AIP	0.63 (0.14)	0.62 (0.14)	0.432
CRI	4.24 (3.36, 5.31)	4.15 (3.30, 5.32)	0.379
TyG	9.12 (0.64)	9.09 (0.65)	0.295
METS-IR	39.32 (34.83, 44.89)	38.62 (34.54, 44.24)	0.058

BMI, body mass index; SBP, systolic blood pressure; DBP, diastolic blood pressure; WBC, white blood cell count; NEU, neutrophil count; EOC, eosinophil count; BASO, basophil count; LYC, lymphocyte count; RBC, red blood cell count; HB, hemoglobin; RDW, red blood cell distribution width; MCV, mean red blood cell volume; PLT, platelet count; PDW, platelet distribution width; MPV, mean platelet volume; TC, total cholesterol; TG, triglycerides; HDL, high-density lipoprotein; LDL, low-density lipoprotein; FBG, fasting blood glucose; TP, total protein; ALB, albumin; AST, aspartate aminotransferase; ALT, alanine aminotransferase; GGT, gamma-glutamyl transpeptidase; SUA, serum uric acid; SCR, serum creatinine; AIP, atherogenic index of plasma; CRI, Castelli risk index; TyG, triglyceride-glucose; METS-IR, metabolic score for insulin resistance.

### 3.4 Validation of risk prediction models

**Figure 7:**
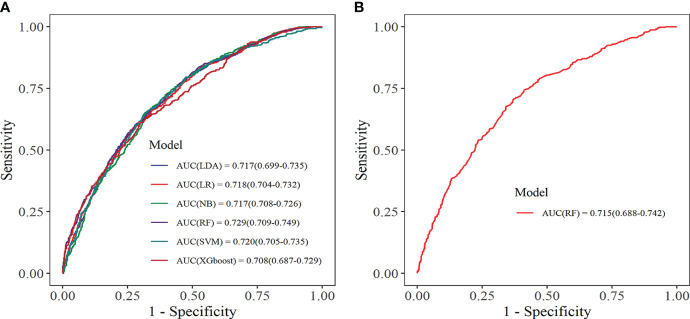
Receiver operating characteristic curve. **(A)** Training set; **(B)** Internal validation set. LDA, linear discriminant analysis; LR, logistic regression; NB, Naive Bayes; RF, random forest; SVM, support vector machine; XGboost, extreme gradient boosting.

**Figure 8:**
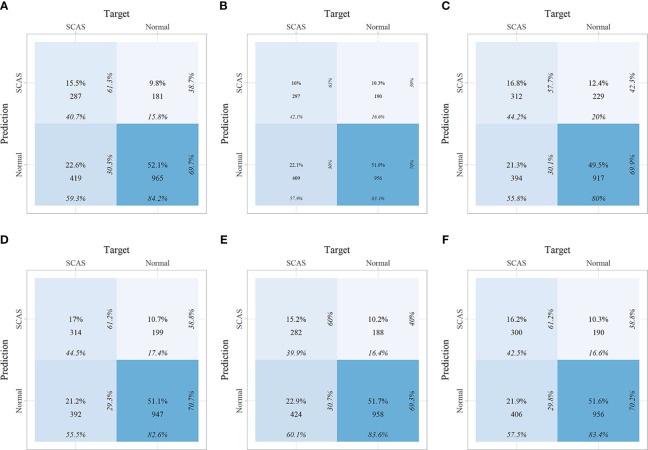
The confusion matrix of the six machine learning models in the training set. **(A)** Linear discriminant analysis; **(B)** Logistic regression; **(C)** Naive Bayes; **(D)** Random forest; **(E)** Support vector machine; **(F)** Extreme gradient boosting.

Within the training set, 10-fold cross-validation was employed to evaluate the predictive value of the models and showed that the RF model had the best clinical predictive value [AUC: 0.729 (95% CI: 0.709-0.749)], followed by the SVM model [AUC: 0.720 (0.705-0.735)](**Figure 7A**). In the internal validation set, the RF model also demonstrated a good clinical predictive value [AUC: 0.715 (95% CI: 0.688-0.742)](**Figure 7B**). Furthermore, a comprehensive comparison of other clinical performance parameters, such as sensitivity and specificity, was conducted among the prediction models (**Table 3**). From the table, we observed that the RF model exhibits excellent performance in various parameters in the training set. The confusion matrix of the six machine learning models in the training set is shown in **Figure 8**.

**Table 3:** Performance parameters of six machine learning prediction models in the training set.

Model	Accuracy	Sensitivity	Specificity	Precision	Recall	F1
LDA	0.676 (0.654-0.697)	0.407	0.842	0.613	0.407	0.489
LR	0.677 (0.655-0.698)	0.421	0.834	0.610	0.421	0.498
NB	0.664 (0.642-0.685)	0.442	0.800	0.577	0.442	0.500
RF	0.681 (0.659-0.702)	0.445	0.826	0.612	0.445	0.515
SVM	0.670 (0.648-0.691)	0.399	0.836	0.600	0.399	0.480
XGboost	0.678 (0.656-0.699)	0.425	0.834	0.612	0.425	0.502

LDA, linear discriminant analysis; LR, logistic regression; NB, Naive Bayes; RF, random forest; SVM, support vector machine; XGboost, extreme gradient boosting.

**Figure 9:**
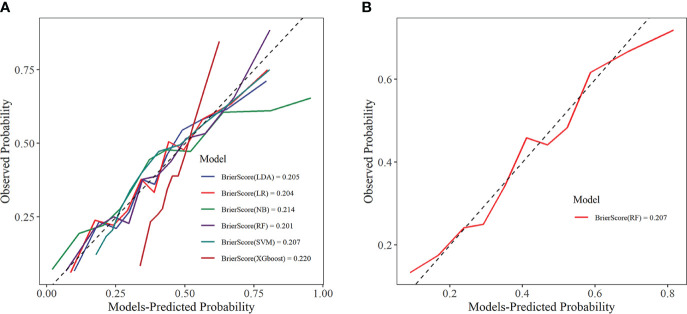
Calibration curve. **(A)** Training set; **(B)** Internal validation set. LDA, linear discriminant analysis; LR, logistic regression; NB, Naive Bayes; RF, random forest; SVM, support vector machine; XGboost, extreme gradient boosting.

The calibration curve visually displays the fit of the risk prediction models. As shown in **Figure 9**, except for the XGboost and NB models, the predicted values of the other models closely match the theoretical values, demonstrating good clinical calibration.

**Figure 10:**
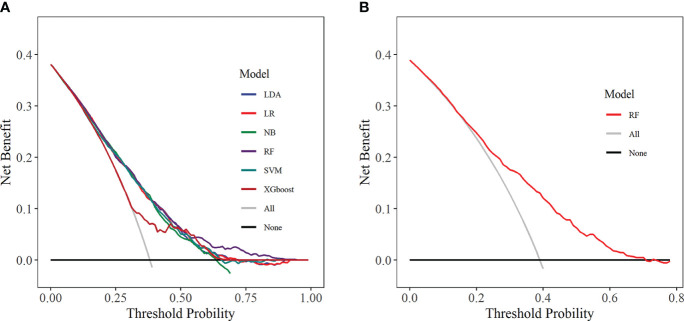
Decision curve analysis. **(A)** Training set; **(B)** Internal validation set. LDA, linear discriminant analysis; LR, logistic regression; NB, Naive Bayes; RF, random forest; SVM, support vector machine; XGboost, extreme gradient boosting.

DCA was used to assess the clinical applicability of predictive models by showing the relationship between risks and benefits corresponding to different decision-making. In the training set, all six ML models showed good clinical applicability (**Figure 10A**). Further, we calculated the risk threshold probability for the RF prediction model in the internal validation set, which showed that the RF model was clinically beneficial in the range of 2%-70% (**Figure 10B**).

### 3.5 Interpretation of risk prediction model

**Figure 11:**
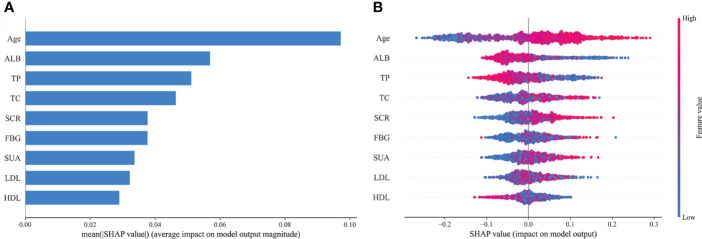
Feature importance of random forest model. **(A)** The importance ranking of the features according to the mean absolute SHAP value; **(B)** The effect of features on the outcome of the model. ALB, albumin; TP, total protein; TC, total cholesterol; SCR, serum creatinine; FBG, fasting blood glucose; SUA, serum uric acid; LDL, low-density lipoprotein; HDL, high-density lipoprotein; SHAP, Shapley Additive exPlanation.

Based on the aforementioned analysis, we found that the RF prediction model demonstrated outstanding performance in both the training and internal validation sets, with the highest clinical predictive value observed in the training set [AUC: 0.729 (95% CI: 0.709-0.749)] and outperformed others in terms of accuracy, sensitivity, recall, and F1 score. Therefore, we have selected the RF model as the optimal prediction model for further model interpretation. SHAP interpretation is currently an emerging and the most commonly used method for interpreting predictive models in the field of ML, which interprets the model by computing the “contribution value” (Shapley values) of each characteristic predictor (21). **Figure 11A** depicts the contribution degree of the characteristic predictors to the prediction model, with the top five variables being Age, ALB, TP, TC, and SCR. Moreover, we observed that higher values of Age, TC, and SCR correspond to higher SHAP values and increased disease risk, whereas higher values of ALB and TP result in smaller SHAP values and reduced disease risk (**Figure 11B**).

## 4 Discussion

This study included a total of 3084 T2DM individuals, of whom 1186 had SCAS. Multivariate logistic regression analysis identified 14 variables, such as Age, WBC, BASO, and LYC (*P* < 0.05) as independent risk factors for SCAS in T2DM patients. LASSO combined with RF-RFE algorithms revealed nine characteristic variables, including Age, FBG, TC, HDL, LDL, TP, ALB, SUA, and SCR, as predictors for the SCAS risk model. Six ML models were developed and validated for clinical performance. Ultimately, the RF model exhibited the highest clinical predictive value in the training set [AUC: 0.729 (0.709-0.749)] and outperformed in accuracy, sensitivity, recall, and F1 score. The SHAP interpretation of the RF model revealed that Age, ALB, TP, TC, and SCR were the top five variables that made the most significant contributions to the predictive model.

In this study, the percentage of SCAS in the T2DM population was 38.46%, lower than the 43.68% reported by Hashimoto et al. in a Japanese T2DM population (22), which might be related to the region and sample size. Multiple studies have demonstrated an association between the TyG index and the incidence of CVD, coronary artery stenosis, stroke, and AS (23, 24). A meta-analysis has revealed that an elevated TyG index is associated with SCAS and arterial stiffness in the adult population (25). Notably, the I-Lan Longitudinal Aging Study identified an association between the TyG index and SCAS in non-diabetic individuals, but not in those with diabetes (26). Consistent with this finding, our study also found no significant statistical association between the TyG index and SCAS in the T2DM population. AIP has emerged as a novel predictive biomarker for CVD. Associations have been identified between elevated AIP levels and increased incidences of CAC and AS (27, 28). In this study, we observed that for every 0.1 unit increase in AIP, the risk of SCAS increased by 0.31-fold [OR: 1.310 (1.201-1.401)]. However, the receiver operating characteristic curve indicated an average diagnostic value for AIP (AUC: 0.535).

Age, PDW, MPV, SUA, and GGT were observed as independent risk factors for SCAS, consistent with previous studies (29–33). Inflammation-related markers such as WBC, BASO, and LYC, were also found to be independent risk factors for SCAS. Long-term studies have shown that AS has a complex pathogenesis, primarily attributed to lipoprotein retention in the arterial wall and chronic inflammation (34, 35). Hyperglycemia leads to increased inflammasome activity, upregulated nucleotide-binding oligomerization domain-like receptor 3, and ultimately elevated pro-inflammatory interleukin1β and interleukin 18 levels (36). Our study further confirms that SCAS in T2DM is a chronic inflammatory condition. Dyslipidemia is a well-established independent risk factor for CVD. In our study, we observed that HDL is an independent risk factor for SCAS. While early research consistently demonstrated an inverse correlation between HDL levels and CVD risk (37, 38), more recent studies have unveiled a non-linear, U-shaped relationship, with very high HDL levels associated with cardiovascular mortality (39, 40).

Optimizing approaches for early diagnosis of SCAS and providing earlier and more precise interventions are crucial to reducing adverse cardiovascular events. Currently, CUS and CT examinations are the primary methods for screening SCAS, but massive generalization inevitably leads to the wastage of medical resources and increased costs, particularly in low-income countries with limited resources. In recent years, with the growing demand for high-quality healthcare, AI has become a powerful tool in clinical medicine. ML, as a branch of AI, was able to analyze large datasets, find complex patterns, and generate insights that contribute to early disease diagnosis, drug discovery, and risk prediction (41, 42). For instance, a study based on electronic health records used ML to generate an in-silico marker for coronary artery disease (CAD) that can non-invasively quantify AS and risk of death on a continuous spectrum, and identify underdiagnosed individuals (43). In addition, Ninomiya et al. (44) developed ML models to predict 5-year all-cause mortality in patients with CAD and assessed ML’s benefit in guiding decision-making between percutaneous coronary intervention (PCI) and coronary artery bypass grafting (CABG). The results showed that the hybrid gradient boosting model was the most effective for predicting 5-year all-cause mortality (C-indexes of 0.78) and that ML is feasible and effective for identifying individuals who benefit from CABG or PCI. In this study, we have developed risk prediction models for SCAS in T2DM patients based on interpretable machine learning methods that could contribute to the early identification of high-risk individuals.

Our study carries significant clinical importance. This might be one of the initial studies to perform SCAS risk prediction in the T2DM population using interpretable ML methods. As a chronic condition, SCAS is challenging to reverse once it develops, emphasizing the effectiveness of early prevention over active treatment. This prediction model enables the identification of high-risk individuals with SCAS within the T2DM population, providing a valuable advantage for early disease prevention. Moreover, the prediction model could bring benefits not only to medically underdeveloped regions but also to inform the clinical decisions of physicians, thus contributing to the optimization of healthcare resources.

This study has certain unavoidable limitations. Firstly, the study population was limited to a specific region, which might impact the generalizability of the prediction model. Secondly, the collection of clinical data lacked comprehensiveness, which may have led to the omission of potential predictors. Thirdly, the risk prediction model has only undergone validation using internal datasets, necessitating further validation with external datasets. In future studies, we will conduct a long-term follow-up study and collaborate with multiple centers to further revise and improve the model.

## 5 Conclusions

In summary, the development, validation, and interpretation of the SCAS risk prediction model in a Chinese T2DM population has significant implications for the reduction and prevention of adverse cardiovascular events.

## Data availability statement

The original contributions presented in the study are included in the article/**Supplementary Material**. Further inquiries can be directed to the corresponding authors.

## Ethics statement

The study protocol adhered to the Declaration of Helsinki and received approval from the Ethics Committee of the Affiliated Hospital of Medical School, Ningbo University, (In March 2023, renamed as The First Affiliated Hospital of Ningbo University), Ningbo, China (KY20220607). Informed consent was obtained from all participants, and the study data were anonymized.

## Author contributions

XT: Data curation, Formal analysis, Methodology, Visualization, Writing – original draft. YS: Data curation, Formal analysis, Methodology, Writing – review & editing. GH: Conceptualization, Data curation, Methodology, Validation, Visualization, Writing – review & editing. YM: Conceptualization, Data curation, Funding acquisition, Supervision, Writing – review & editing.

## References

[1] Sun H Saeedi P Karuranga S Pinkepank M Ogurtsova K Duncan BB. IDF Diabetes Atlas: Global, regional and country-level diabetes prevalence estimates for 2021 and projections for 2045. Diabetes Res Clin Pract (2022) 183:109119. doi: 10.1016/j.diabres.2021.109119 34879977 PMC11057359

[2] Einarson TR Acs A Ludwig C Panton UH. Prevalence of cardiovascular disease in type 2 diabetes: a systematic literature review of scientific evidence from across the world in 2007-2017. Cardiovasc Diabetol (2018) 17:83. doi: 10.1186/s12933-018-0728-6 29884191 PMC5994068

[3] Tsao CW Aday AW Almarzooq ZI Anderson CAM Arora P Avery CL. Heart disease and stroke statistics-2023 update: A report from the American heart association. Circulation (2023) 147:e93–e621. doi: 10.1161/cir.0000000000001123 36695182 PMC12135016

[4] Sarwar N Gao P Seshasai SR Gobin R Kaptoge S Di Angelantonio E. Diabetes mellitus, fasting blood glucose concentration, and risk of vascular disease: A collaborative meta-analysis of 102 prospective studies. Lancet (2010) 375:2215–22. doi: 10.1016/s0140-6736(10)60484-9 PMC2904878 20609967

[5] Gregg EW Sattar N Ali MK. The changing face of diabetes complications. Lancet Diabetes Endocrinol (2016) 4:537–47. doi: 10.1016/s2213-8587(16)30010-9 27156051

[6] Singh SS Pilkerton CS Shrader CD Jr. Frisbee SJ. Subclinical atherosclerosis, cardiovascular health, and disease risk: Is there a case for the Cardiovascular Health Index in the primary prevention population? BMC Public Health (2018) 18:429. doi: 10.1186/s12889-018-5263-6 29609588 PMC5880087

[7] Zaid M Fujiyoshi A Kadota A Abbott RD Miura K. Coronary artery calcium and carotid artery intima media thickness and plaque: Clinical use in need of clarification. J Atheroscler Thromb (2017) 24:227–39. doi: 10.5551/jat.RV16005 PMC5383538 27904029

[8] Jeevarethinam A Venuraju S Dumo A Ruano S Mehta VS Rosenthal M. Relationship between carotid atherosclerosis and coronary artery calcification in asymptomatic diabetic patients: A prospective multicenter study. Clin Cardiol (2017) 40:752–8. doi: 10.1002/clc.22727 PMC6490331 28543093

[9] Prakash S Balaji JN Joshi A Surapaneni KM. Ethical conundrums in the application of artificial intelligence (AI) in healthcare-A scoping review of reviews. J Pers Med (2022) 12:1914. doi: 10.3390/jpm12111914 36422090 PMC9698424

[10] Sánchez-Cabo F Rossello X Fuster V Benito F Manzano JP Silla JC. Machine learning improves cardiovascular risk definition for young, asymptomatic individuals. J Am Coll Cardiol (2020) 76:1674–85. doi: 10.1016/j.jacc.2020.08.017 33004133

[11] Núñez E Fuster V Gómez-Serrano M Valdivielso JM Fernández-Alvira JM Martínez-López D. Unbiased plasma proteomics discovery of biomarkers for improved detection of subclinical atherosclerosis. EBioMedicine (2022) 76:103874. doi: 10.1016/j.ebiom.2022.103874 35152150 PMC8844841

[12] Peduzzi P Concato J Kemper E Holford TR Feinstein AR. A simulation study of the number of events per variable in logistic regression analysis. J Clin Epidemiol (1996) 49:1373–9. doi: 10.1016/s0895-4356(96)00236-3 8970487

[13] ElSayed NA Aleppo G Aroda VR Bannuru RR Brown FM Bruemmer D. Addendum. 2. Classification and diagnosis of diabetes: Standards of care in diabetes-2023. Diabetes Care (2023) 46:S19–40. doi: 10.2337/dc23-ad08 PMC10552401 37356047

[14] Wu L Qian L Zhang L Zhang J Zhou J Li Y. Fibroblast growth factor 21 is related to atherosclerosis independent of nonalcoholic fatty liver disease and predicts atherosclerotic cardiovascular events. J Am Heart Assoc (2020) 9:e015226. doi: 10.1161/jaha.119.015226 32431189 PMC7428997

[15] Lioy B Webb RJ Amirabdollahian F. The association between the atherogenic index of plasma and cardiometabolic risk factors: A review. Healthcare (Basel) (2023) 11:966. doi: 10.3390/healthcare11070966 37046893 PMC10094587

[16] Mahdavi-Roshan M Mozafarihashjin M Shoaibinobarian N Ghorbani Z Salari A Savarrakhsh A. Evaluating the use of novel atherogenicity indices and insulin resistance surrogate markers in predicting the risk of coronary artery disease: A case−control investigation with comparison to traditional biomarkers. Lipids Health Dis (2022) 21:126. doi: 10.1186/s12944-022-01732-9 36435770 PMC9701407

[17] Zhang X Liu F Li W Zhang J Zhang T Yu X. Metabolic score for insulin resistance (METS-IR) predicts adverse cardiovascular events in patients with type 2 diabetes and ischemic cardiomyopathy. Diabetes Metab Syndr Obes (2023) 16:1283–95. doi: 10.2147/dmso.S404878 PMC10167964 37179787

[18] Thai PV Tien HA Van Minh H Valensi P. Triglyceride glucose index for the detection of asymptomatic coronary artery stenosis in patients with type 2 diabetes. Cardiovasc Diabetol (2020) 19:137. doi: 10.1186/s12933-020-01108-2 32919465 PMC7488689

[19] Zheng Z Si Z Wang X Meng R Wang H Zhao Z. Risk prediction for the development of hyperuricemia: Model development using an occupational health examination dataset. Int J Environ Res Public Health (2023) 20:3411. doi: 10.3390/ijerph20043411 36834107 PMC9967697

[20] Zhou L Wang Q Yin P Xing W Wu Z Chen S. Serum metabolomics reveals the deregulation of fatty acids metabolism in hepatocellular carcinoma and chronic liver diseases. Anal Bioanal Chem (2012) 403:203–13. doi: 10.1007/s00216-012-5782-4 22349331

[21] Linardatos P Papastefanopoulos V Kotsiantis S. Explainable AI: A review of machine learning interpretability methods. Entropy (Basel) (2020) 23:18. doi: 10.3390/e23010018 33375658 PMC7824368

[22] Hashimoto Y Takahashi F Okamura T Osaka T Okada H Senmaru T. Relationship between serum creatinine to cystatin C ratio and subclinical atherosclerosis in patients with type 2 diabetes. BMJ Open Diabetes Res Care (2022) 10:e002910. doi: 10.1136/bmjdrc-2022-002910 PMC9226914 35738823

[23] Li H Jiang Y Su X Meng Z. The triglyceride glucose index was U-shape associated with all-cause mortality in population with cardiovascular diseases. Diabetol Metab Syndr (2023) 15:181. doi: 10.1186/s13098-023-01153-3 37679825 PMC10483863

[24] da Silva A Caldas APS Hermsdorff HHM Bersch-Ferreira ÂC Torreglosa CR Weber B. Triglyceride-glucose index is associated with symptomatic coronary artery disease in patients in secondary care. Cardiovasc Diabetol (2019) 18:89. doi: 10.1186/s12933-019-0893-2 31296225 PMC6625050

[25] Sajdeya O Beran A Mhanna M Alharbi A Burmeister C Abuhelwa Z. Triglyceride glucose index for the prediction of subclinical atherosclerosis and arterial stiffness: A meta-analysis of 37,780 individuals. Curr Probl Cardiol (2022) 47:101390. doi: 10.1016/j.cpcardiol.2022.101390 36103942

[26] Lu YW Chang CC Chou RH Tsai YL Liu LK Chen LK. Gender difference in the association between TyG index and subclinical atherosclerosis: Results from the I-Lan Longitudinal Aging Study. Cardiovasc Diabetol (2021) 20:206. doi: 10.1186/s12933-021-01391-7 34645432 PMC8515653

[27] Nam JS Kim MK Nam JY Park K Kang S Ahn CW. Association between atherogenic index of plasma and coronary artery calcification progression in Korean adults. Lipids Health Dis (2020) 19:157. doi: 10.1186/s12944-020-01317-4 32615982 PMC7331149

[28] Huang Q Liu Z Wei M Huang Q Feng J Liu Z. The atherogenic index of plasma and carotid atherosclerosis in a community population: A population-based cohort study in China. Cardiovasc Diabetol (2023) 22:125. doi: 10.1186/s12933-023-01977-3 37244995 PMC10225098

[29] van den Munckhof ICL Jones H Hopman MTE de Graaf J Nyakayiru J van Dijk B. Relation between age and carotid artery intima-medial thickness: a systematic review. Clin Cardiol (2018) 41:698–704. doi: 10.1002/clc.22934 PMC6489962 29752816

[30] Lappegård J Ellingsen TS Vik A Skjelbakken T Brox J Mathiesen EB. Red cell distribution width and carotid atherosclerosis progression. Tromsø Study. Thromb Haemost (2015) 113:649–54. doi: 10.1160/th14-07-0606 25631329

[31] Adam G Kocak E Reşorlu M. Evaluation of platelet distribution width and mean platelet volume in patients with carotid artery stenosis: Author's reply. Angiology (2015) 66:380. doi: 10.1177/0003319714565169 25313243

[32] Gao Y Xu B Yang Y Zhang M Yu T Zhang Q. Association between serum uric acid and carotid intima-media thickness in different fasting blood glucose patterns: A case-control study. Front Endocrinol (Lausanne) (2022) 13:899241. doi: 10.3389/fendo.2022.899241 35712254 PMC9197240

[33] Kim YG Park GM Lee SB Yang DH Kang JW Lim TH. Association of gamma-glutamyl transferase with subclinical coronary atherosclerosis and cardiac outcomes in non-alcoholics. Sci Rep (2020) 10:17994. doi: 10.1038/s41598-020-75078-6 33093619 PMC7581814

[34] Wojtasińska A Frąk W Lisińska W Sapeda N Młynarska E Rysz J. Novel insights into the molecular mechanisms of atherosclerosis. Int J Mol Sci (2023) 24:13434. doi: 10.3390/ijms241713434 37686238 PMC10487483

[35] Morrison AM Sullivan AE Aday AW. Atherosclerotic disease: Pathogenesis and approaches to management. Med Clin North Am (2023) 107:793–805. doi: 10.1016/j.mcna.2023.04.004 37541708 PMC10547111

[36] Alfadul H Sabico S Ansari MGA Alnaami AM Amer OE Hussain SD. Differences and associations of NLRP3 inflammasome levels with interleukins 1α, 1β, 33 and 37 in adults with prediabetes and type 2 diabetes mellitus. Biomedicines (2023) 11:1315. doi: 10.3390/biomedicines11051315 37238986 PMC10216290

[37] Gordon DJ Probstfield JL Garrison RJ Neaton JD Castelli WP Knoke JD. High-density lipoprotein cholesterol and cardiovascular disease. Four prospective Am Stud Circulation (1989) 79:8–15. doi: 10.1161/01.CIR.79.1.8 2642759

[38] Ko DT Alter DA Guo H Koh M Lau G Austin PC. High-density lipoprotein cholesterol and cause-specific mortality in individuals without previous cardiovascular conditions: The CANHEART study. J Am Coll Cardiol (2016) 68:2073–83. doi: 10.1016/j.jacc.2016.08.038 27810046

[39] Madsen CM Varbo A Nordestgaard BG. Extreme high high-density lipoprotein cholesterol is paradoxically associated with high mortality in men and women: two prospective cohort studies. Eur Heart J (2017) 38:2478–86. doi: 10.1093/eurheartj/ehx163 28419274

[40] Liu C Dhindsa D Almuwaqqat Z Ko YA Mehta A Alkhoder AA. Association between high-density lipoprotein cholesterol levels and adverse cardiovascular outcomes in high-risk populations. JAMA Cardiol (2022) 7:672–80. doi: 10.1001/jamacardio.2022.0912 PMC9118072 35583863

[41] Kumar Y Koul A Singla R Ijaz MF. Artificial intelligence in disease diagnosis: A systematic literature review, synthesizing framework and future research agenda. J Ambient Intell Humaniz Comput (2023) 14:8459–86. doi: 10.1007/s12652-021-03612-z PMC8754556 35039756

[42] Krishnan G Singh S Pathania M Gosavi S Abhishek S Parchani A. Artificial intelligence in clinical medicine: Catalyzing a sustainable global healthcare paradigm. Front Artif Intell (2023) 6:1227091. doi: 10.3389/frai.2023.1227091 37705603 PMC10497111

[43] Forrest IS Petrazzini BO Duffy Á Park JK Marquez-Luna C Jordan DM. Machine learning-based marker for coronary artery disease: Derivation and validation in two longitudinal cohorts. Lancet (2023) 401:215–25. doi: 10.1016/s0140-6736(22)02079-7 PMC10069625 36563696

[44] Ninomiya K Kageyama S Shiomi H Kotoku N Masuda S Revaiah PC. Can machine learning aid the selection of percutaneous vs surgical revascularization? J Am Coll Cardiol (2023) 82:2113–24. doi: 10.1016/j.jacc.2023.09.818 37993203

