# Supplementary material for: Predicting the risk of subclinical atherosclerosis based on interpretable machine models in a Chinese T2DM population
Source: Front Endocrinol (Lausanne). 2024 Feb 27;15:1332982. doi: 10.3389/fendo.2024.1332982 (PMC10929018; doi:10.3389/fendo.2024.1332982)
Supplement: Supplementary file 1 [file Image_1.pdf]

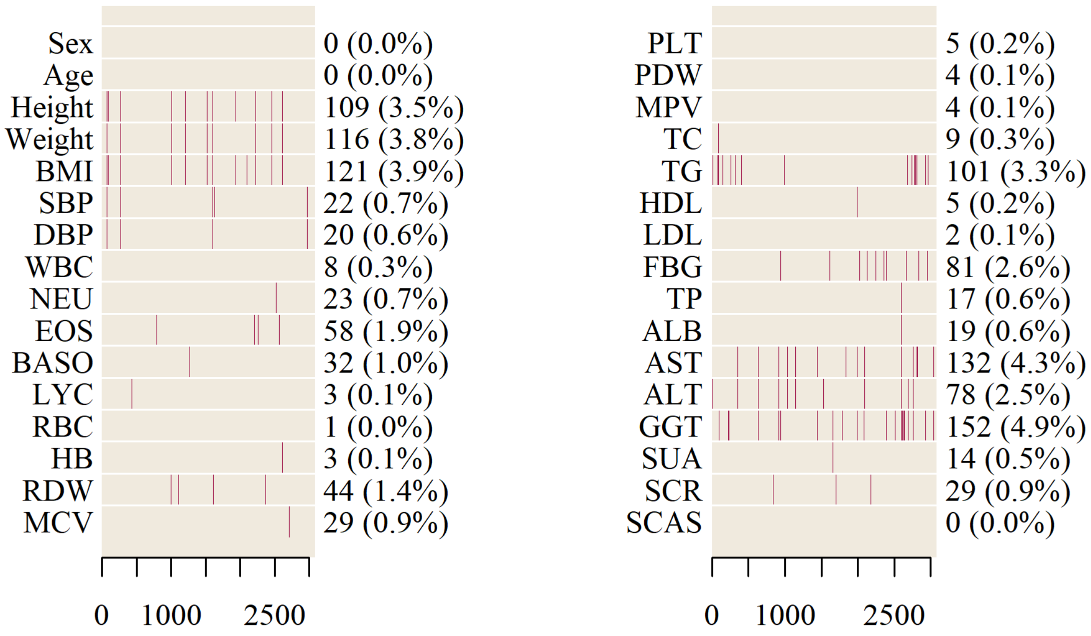

Figure S1. The distribution of the missing data. BMI, body mass index; SBP, systolic blood pressure; DBP, diastolic blood pressure; WBC, white blood cell count; NEU, neutrophil count; EOC, eosinophil count; BASO, basophil count; LYC, lymphocyte count; RBC, red blood cell count; HB, hemoglobin; RDW, red blood cell distribution width; MCV, mean red blood cell volume; PLT, platelet count; PDW, platelet distribution width; MPV, mean platelet volume; TC, total cholesterol; TG, triglycerides; HDL, high-density lipoprotein; LDL, low-density lipoprotein; FBG, fasting blood glucose; TP, total protein; ALB, albumin; AST, aspartate aminotransferase; ALT, alanine aminotransferase; GGT, gamma-glutamyl transpeptidase; SUA, serum uric acid; SCR, serum creatinine; SCAS, subclinical atherosclerosis.

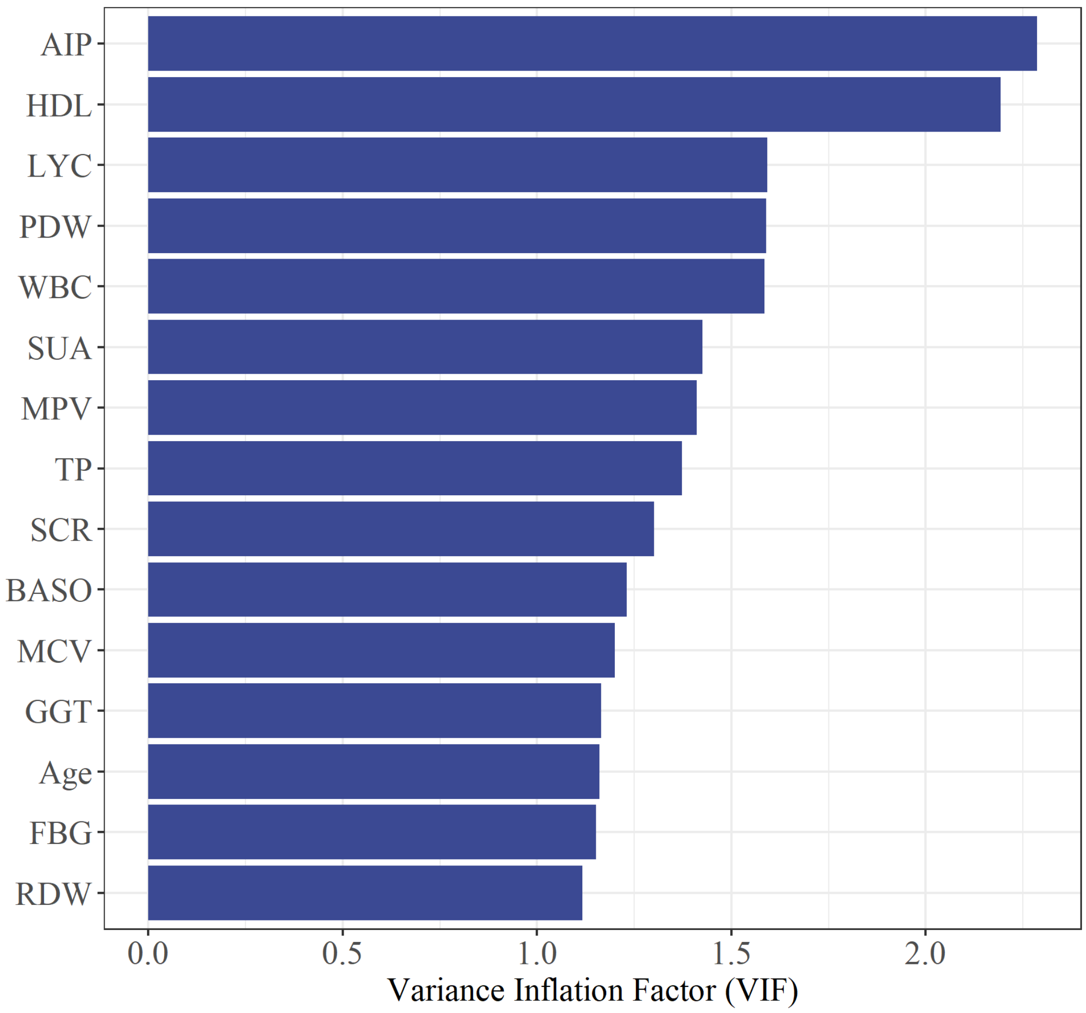

Figure S2. Variance inflation factors for candidate independent risk factors. AIP, atherogenic index of plasma; HDL, high-density lipoprotein; LYC, lymphocyte count; PDW, platelet distribution width; WBC, white blood cell count; SUA, serum uric acid; MPV, mean platelet volume; TP, total protein; SCR, serum creatinine; BASO, basophil count; MCV, mean red blood cell volume; GGT, gamma-glutamyl transpeptidase; FBG, fasting blood glucose; RDW, red blood cell distribution width.
